# Antenatal diagnosis of large cervical mass in foetus

**DOI:** 10.11604/pamj.2024.49.99.43821

**Published:** 2024-11-28

**Authors:** Neha Hemant Chhajed, Neelam Hemant Chhajed

**Affiliations:** 1 Datta Meghe Medical College, Datta Meghe Institute of Higher Education and Research, Wardha, Maharashtra 442107, India,; 2 New Life Hospital & Fetal Medicine Centre, Nagpur, Nagpur, Maharashtra, India

**Keywords:** Cervical teratoma, hemangioma, hamartoma

## Image in medicine

We report the case of cervical mass in foetus which was antenatally diagnosed during the second trimester. The mother was primigravida, with no history of teratogenic drug consumption, had no previously acquired TORCH infections and was hemodynamically stable. The cervical mass appeared to be progressively growing in size from 6.76 x 5.05 cm to 10.92 x 8.60 cm which we continuously monitored with successive growth scans throughout the pregnancy. An ex-utero intrapartum treatment (EXIT) procedure i.e. ex-utero intrapartum treatment was planned. However, it was not required as no features of airway obstruction were noted immediately after delivery and the baby was born with good Apgar scores of 9 to 10 per minute. On day 3 post-birth an elective mass resection surgery was performed wherein the mass was aspirated and partially removed. Some of the mass tissue was left in situ as it was engulfing the carotid artery and was too dangerous to be excised. On day 2 post-operative, the baby was extubated and found to have normal respiratory parameters. On day 7, the baby was discharged. Although antenatal diagnosis pointed towards the mass being cervical teratoma because of suspected calcification, histopathological findings confirmed the diagnosis of a cystic hygroma.

**Figure 1:**
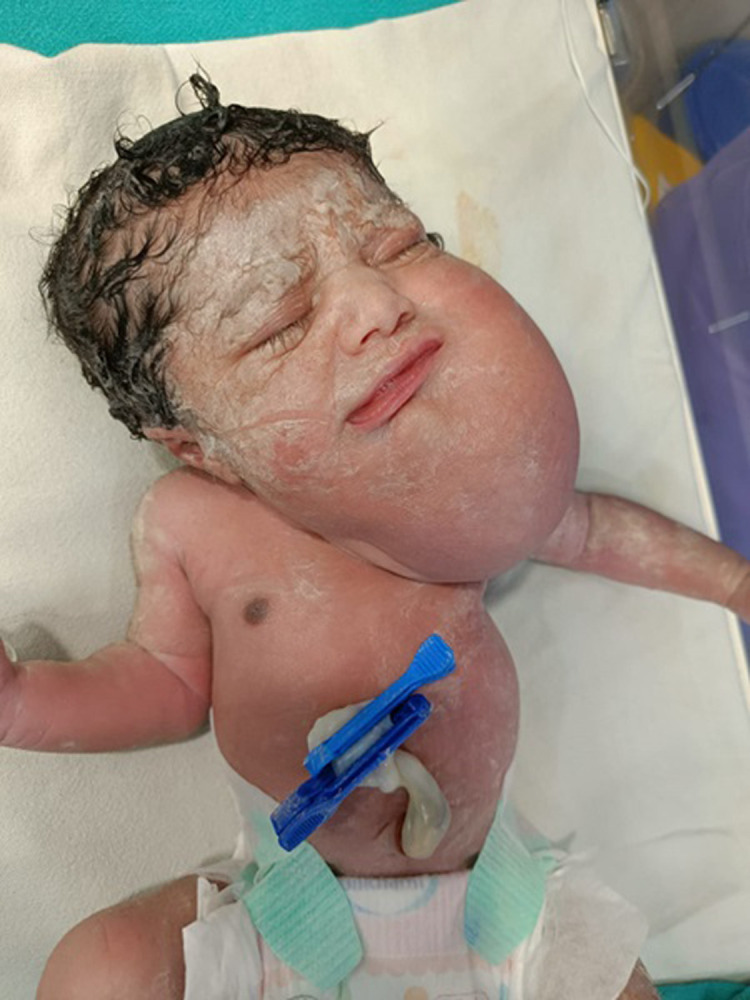
baby at birth with cystic hygroma

